# A novel approach to evaluate the effects of artificial bone focal lesion on the three-dimensional strain distributions within the vertebral body

**DOI:** 10.1371/journal.pone.0251873

**Published:** 2021-06-01

**Authors:** Marco Palanca, Giulia De Donno, Enrico Dall’Ara

**Affiliations:** 1 Dept of Oncology and Metabolism and INSIGNEO Institute for *in silico* Medicine, The University of Sheffield, Sheffield, United Kingdom; 2 Dept of Industrial Engineering, Alma Mater Studiorum, Università di Bologna, Bologna, Italy; Medical College of Wisconsin, UNITED STATES

## Abstract

The spine is the first site for incidence of bone metastasis. Thus, the vertebrae have a high potential risk of being weakened by metastatic tissues. The evaluation of strength of the bone affected by the presence of metastases is fundamental to assess the fracture risk. This work proposes a robust method to evaluate the variations of strain distributions due to artificial lesions within the vertebral body, based on *in situ* mechanical testing and digital volume correlation. Five porcine vertebrae were tested in compression up to 6500N inside a micro computed tomography scanner. For each specimen, images were acquired before and after the application of the load, before and after the introduction of the artificial lesions. Principal strains were computed within the bone by means of digital volume correlation (DVC). All intact specimens showed a consistent strain distribution, with peak minimum principal strain in the range -1.8% to -0.7% in the middle of the vertebra, demonstrating the robustness of the method. Similar distributions of strains were found for the intact vertebrae in the different regions. The artificial lesion generally doubled the strain in the middle portion of the specimen, probably due to stress concentrations close to the defect. In conclusion, a robust method to evaluate the redistribution of the strain due to artificial lesions within the vertebral body was developed and will be used in the future to improve current clinical assessment of fracture risk in metastatic spines.

## Introduction

The spine has high incidence of bone metastases [1, 2]. The evaluation of the biomechanical stability of vertebrae with metastases is fundamental to identify patients at high risk of fracture and to develop clinical tools for optimising and personalising their treatment.

Different studies have evaluated the effect of metastatic lesions on the vertebral mechanics, in order to prevent fractures. Experimental tests were performed on healthy vertebrae with artificial lesions (usually reproduced as holes in the structure) [3–7] and vertebrae with actual metastasis [8]. The results showed reduced apparent properties of the vertebrae with lesions (failure load and stiffness). Moreover, strain measurements were performed with strain gauges (human vertebrae, induced lesions) [4, 9], digital image correlation (human vertebrae, induced lesions) [5, 7] and digital volume correlation (DVC, small rodents, actual induced metastasis [10]).

While detailed experimental measurements can provide important insights on the biomechanical stability of the vertebrae with lesions, they suffer of two main limitations: the intrinsic inability of testing the same vertebra with/without lesions, and the lack of an approach to evaluate their effect on local deformations in the internal structure of the vertebral body in mammals.

Equivalent efforts to evaluate the effect of metastatic lesions on structural and local properties were made by means of finite element (FE) models. Generic [9, 11–13] or micro-FE [14] models were used to estimate the effect of the independent features of the artificial metastasis (i.e. dimensions, positions, etc.) on the vertebral mechanics. In other studies computed tomography (CT)-based subject-specific homogenised FE models of vertebrae with actual metastases were developed to study the stability of the vertebra [4, 14–16]. Nevertheless, only two of the mentioned studies were validated against experimental measurements of local strain with strain-gauges [4, 9]. Considering the complexity of the internal structure of the vertebra, performing a validation in a few localised regions on the cortical shell is not sufficient to understand if the models are able to predict the strain field within the whole geometry. Therefore, a more comprehensive validation of the predictions of internal displacement and strain fields should be performed by using fields methods like DVC [17]. The experimental and computational approaches have reported conflicting conclusions about the relevance of lesion size and position on the mechanical properties of the vertebral body, probably due to methodological differences in the studies (e.g. loading conditions, measurements, density and morphology of the input vertebrae) and the lack of validation of the FE models.

Thereby, a reliable experimental procedure able to generate reproducible loading conditions and to measure and analyse the 3D strain field in the whole vertebral body would be an invaluable tool to understand the strain redistribution associated to the features of the metastasis. The DVC approach has been intensively used to evaluate the biomechanical properties of the vertebrae [18–20] and to evaluate the accuracy of the FE models in predicting the local displacements and strains in the vertebral body [17, 21, 22].

The aim of this study was to develop a reproducible experimental methodology, based on *in situ* mechanical testing and DVC measurements, to evaluate the local strain distribution within the whole vertebral body and in specific subregions when an artificial lesion was introduced.

## Materials and methods

Porcine spine segments were tested in a step-wise loading within a micro computed tomography (microCT) scanner. Images were acquired with the specimens unloaded and loaded, before and after an artificial lesion was induced. A global DVC approach was used to experimentally measure the effect of the lesion on the displacement and strain fields.

### Sample preparation and preparation of the artificial focal lesion

Five thoracic spinal segments (three vertebrae each) were isolated from two spines of female pigs (nine months old and approximately 100kg in weight) killed for alimentary purposes: two T8-T10, two T10-T12 and one T12-T14. The ligaments, muscles and posterior arches were removed from the spines. When not prepared or tested the specimens were stored frozen at -20C. The segments were aligned as reported in [23]. The caudal half of the most caudal vertebra and the cranial half of the most cranial vertebra were embedded in parallel poly-methyl-methacrylate (Technovit, Germany) bases that were fixed to a custom made loading device, in order to load the middle vertebra through the intervertebral discs [19, 24].

Artificial mechanical lesions were induced to simulate what happens in case of a vertebra with lytic metastatic lesions. The lesions were reproduced in the mid-transverse plane of the middle vertebra of each segment, as holes involving the trabecular bone and cortical shell [5, 14, 25–27].

**Fig 1:**
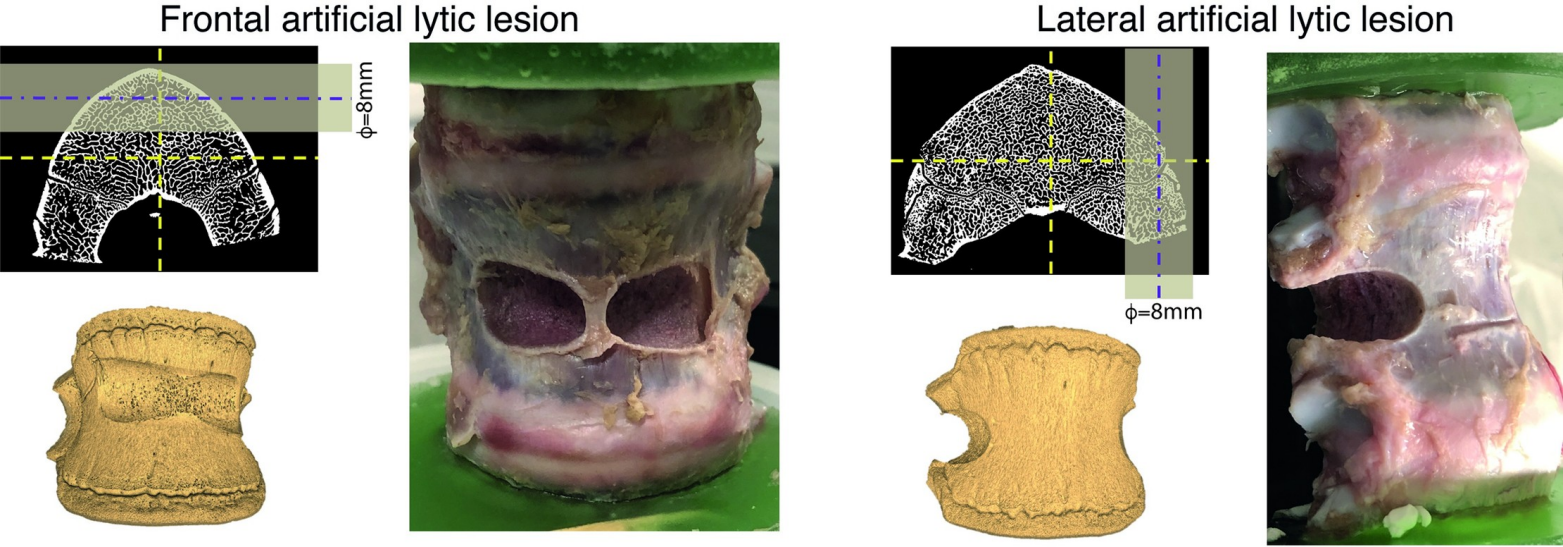
Artificial lesions were reproduced in the anterior or lateral middle portion of the middle. The position and volume of the lesions were identified in the microCT images.

A pillar drill with a diamond core tool 8mm in diameter was used to produce the hole under constant water irrigation to avoid overheating the bone. Two lesion locations were selected to evaluate the procedure in different scenarios: the anterior side (specimen #1, #2, #3) or the right side (specimen #4, #5). In both cases, lesions with approximately half cylindrical shape were created (Fig 1). The position and volume of the lesions were measured from the microCT images. Moreover, the features of the lesion did not reflect the actual metastatic lesion, but they provided a comparable mechanical effect [27].

### Loading condition

A custom-made loading device equipped with a 10kN load cell (HBM, Germany), a 20 mm linear variable differential transformed (LVDT) transducer (HBM, Germany), and an external Perspex tube was used to axially compress the specimen inside a microCT scanner (VivaCT80, Scanco Medical, Switzerland)[28]. Each specimen was wrapped with gauzes soaked in saline solution, fixed in the jig to be loaded, and microCT scanned. The loads were manually applied through a screw with a ratchet wrench and the torsional component of the load was removed with a thrust bearing. To define a loading condition able to generate relevant strains within the intact vertebrae without damaging the tissue, a preliminary work was performed on specimen #1 (the entire procedures and results were reported in detail in the S1 Appendix). Briefly, the specimen was loaded with four different load magnitudes (1500, 3000, 4500, 6500 N), and a load of 6500N, which was consistent with previous literature [20], was considered optimal for the purpose of this study. Ten compressive pre-cycles between 50N and 300N were applied to precondition the specimen, followed by the application of a displacement up to reach a compressive load of 6500N. After that, no additional displacement was imposed during the test. After each load-step, the specimen was left to relax for 15 minutes in order to reach a stable load. After this relaxation time, the acquisition of the microCT images started. The same loading procedure was applied to the vertebrae before and after the creation of the lesion.

### Scanning procedure and image pre-processing

Every scan was acquired with the following parameters: current 114mA, voltage 70kVp, integration time 300ms, power 8W, isotropic voxel size 39μm. The images were reconstructed using the manufacturer software with a beam hardening correction based on a phantom with 1200 mg HA/cm^3^ density [29]. A similar procedure was previously used to scan human vertebrae [14].

**Fig 2:**
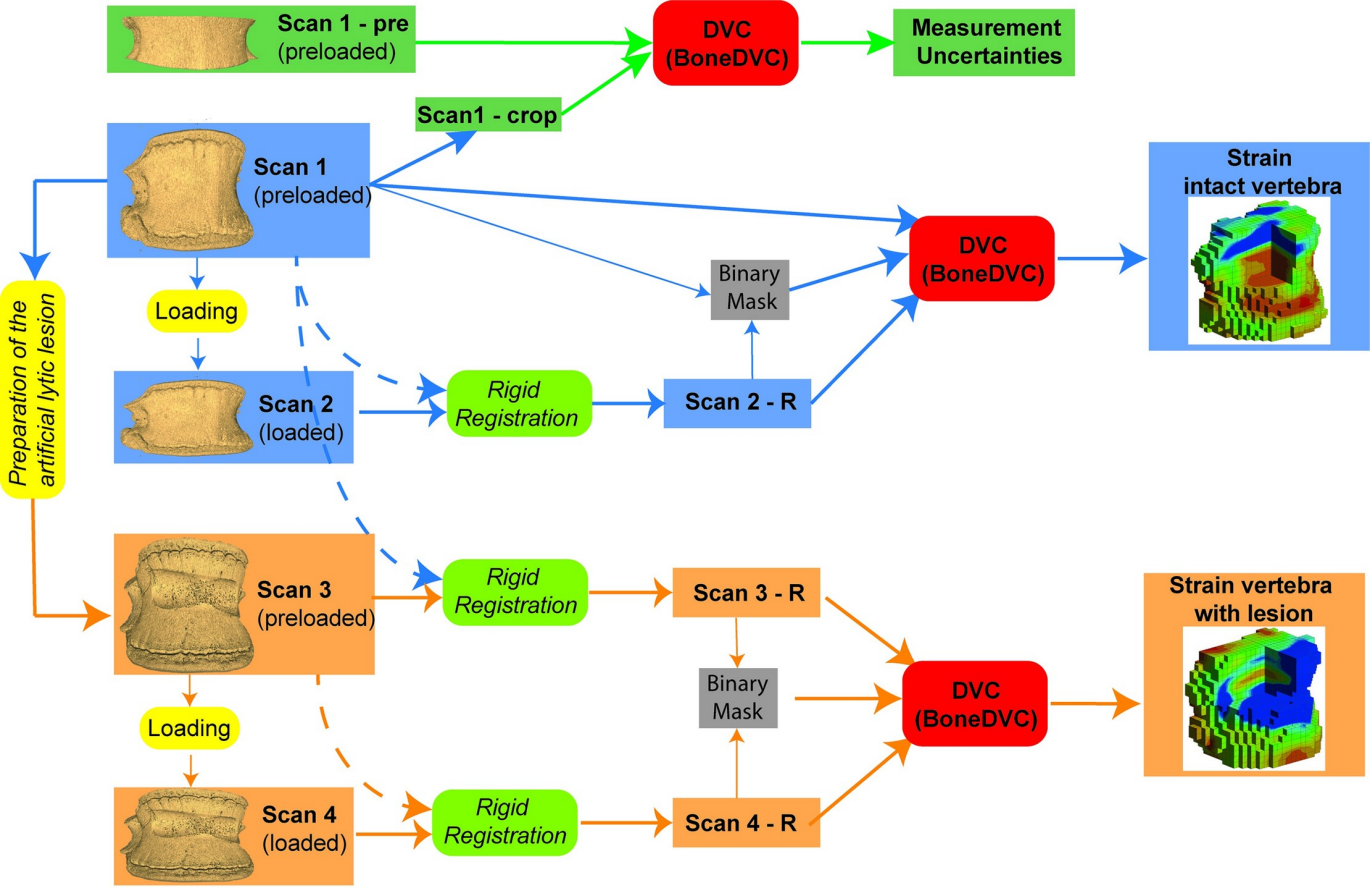
Workflow of the image processing. For each specimen, five scans were acquired to evaluate: the measurement uncertainties (Scan1-pre vs Scan1-crop), the strain field within the vertebral body of the intact vertebra (Scan1 vs Scan2), and the strain field within the vertebral body of the vertebra with artificial lesion (Scan3 vs Scan4). Pre-processing of the images, digital volume correlation (DVC) with the BoneDVC algorithm were used to measure the strain fields. A comparison between the two conditions was proposed.

The middle vertebra of each spine unit (except for specimen #1, see S1 Appendix) was scanned five times (Fig 2):

- “Scan1-pre”: scan of a middle portion of the vertebra in preloaded (approximately 50N) condition. This scan was used together with Scan1 to evaluate the DVC strain measurement uncertainties;
- “Scan1” and “Scan2”: scans of the intact vertebra in preloaded (approximately 50N, Scan1) and loaded (approximately 6500N, Scan2) conditions, respectively. These scans were used to evaluate the strain field in the intact vertebral body;
- “Scan3” and “Scan4”: scans of the vertebra with artificial lesion in preloaded (approximately 50N, Scan3) and loaded (approximately 6500N, Scan4) conditions, respectively. These scans were used to evaluate the strain field in the vertebral body with the lesion.

The scanning time was approximately 66.7 min for the entire vertebral body, and 13.3 min for the middle portions of the vertebra (Scan1-pre). A portion of the Scan1 (Scan1-crop) with the same size and position of Scan1-pre was extracted and used for evaluating the DVC measurement uncertainties.

To evaluate the strain distributions in the same location and with the same orientation of the vertebral body, in both tests with and without the lesion, Scan3 was rigidly registered (Amira 6.2.0, Thermo Fisher Scientific, USA; alignment of principal axes; Lanczos interpolation) to Scan1 (later referred to as Scan3-R). A similar procedure was used to rigidly register the images before and after the load for the elastic registration: Scan2 and Scan4 were registered to Scan1 and Scan3-R, respectively (later referred to as Scan2-R and Scan4-R). This procedure enabled to align all specimens to Scan1, improving the rigid registration between images with similar features (i.e. the drilled holes) before and after the load. Binary masks (zero outside the external contour of the vertebral body, one inside it) were created for each image using a low threshold value, followed by dilation (3 voxels) and by filling the internal holes (ImageJ-Rasband, W.S., ImageJ, U.S. National Institutes of Health, Bethesda, Maryland, USA, https://imagej.nih.gov/ij/, 1997–2018). For each couple of preloaded and loaded images, a mask created by merging the masks obtained from the images of the vertebra in the preloaded and loaded conditions was used for the DVC analyses (registration only within the mask). The preloaded mask was used for visualising only the results within the vertebral body.

The interested reader can access the images used in this study at https://doi.org/10.15131/shef.data.14554842 or by contacting the corresponding author.

### Digital volume correlation

A global DVC approach (BoneDVC, bonedvc.insigneo.org/dvc) was used to measure the strain field inside the vertebrae with and without the created lesions, given the microCT images acquired in the preloaded and loaded conditions. The operating principles of the DVC are reported in details elsewhere [30–32]. Briefly, the images of the preloaded and loaded vertebrae were elastically registered (Sheffield Image Registration Toolkit, ShIRT) to compute the displacement field. The registration algorithm consists in superimposing a regular parallelepiped grid with cubic cells with side length equal to the nodal spacing (NS). ShIRT solves the registration equations in the nodes of the grid within the combined mask. The registration equations include the displacement terms and a term to account for potential changes in the grey levels, trilinear interpolation is assumed within the cells of the grid. The registration is solved by adding a smoothing coefficient in the displacement field to overcome the poorly conditioned problem. The problem is then solved iteratively to compensate for large displacements [32]. The strain field (components of strain, maximum principal strain ε_p1_ and minimum principal strain ε_p3_) is obtained differentiating the displacement field with an FE software package (Mechanical APDL v19, ANSYS, USA) [33]. To do so, a linear hexahedral mesh equal to the hexahedral DVC grid was imported in the FE software. The displacement calculated from the elastic registration in each node of the grid were imposed at the nodes of the FE elements. The displacements were then differentiated into strains. The FE software package was used to visualize the results. Finally, all cells of the DVC grid without any node within the binary mask of the image of the preloaded vertebra were removed to exclude strain values outside the vertebra [34]. This DVC approach has been intensively used to evaluate the displacement and deformation in different bone structures (e.g. trabecular bone [35], porcine vertebrae [17], mouse tibia [36], human scapula [37], proximal humeral head [38] and femoral head [39]). While the DVC approach has a large potential, it is fundamental to optimise the value of the NS for each application by evaluating the uncertainties of the approach [31–33, 40]. The NS was optimised by analysing the strain uncertainties from repeated scans (zero-strain condition) for one specimen with different NS (ranging from 10 to 90 voxels) (The results were reported in the S1 Appendix). A NS of 50 voxels (experimental measurement spatial resolution = 1.95mm) led to a Standard Deviation of the Error (SDER) equal to 148με which was considered acceptable for the considered application. Moreover, considering the variability of the DVC approaches for the local structure [41], the strain measurement uncertainties were estimated for each specimen for NS of 50 voxels, which provided an average SDER equal to 337 με among the five considered specimens (All results were reported in the S1 Appendix).

### Comparison between intact and with focal lesions vertebrae

The ε_p1_ and ε_p3_ were measured for the intact vertebrae (Scan1 vs Scan2-R) and for the vertebrae with artificial lesion (Scan3-R vs Scan4-R). Frequency plots (histogram) and the range of median of the ε_p1_ and ε_p3_ was calculated to assess the repeatability of the loading condition and strain distribution for the intact vertebrae. Strain distributions and histograms for the ε_p1_ (in the S2 Appendix) and ε_p3_ were reported to highlight the changes in strain pattern due to the presence of the lesion.

Each vertebra was divided into three longitudinal regions of interest (ROIs): top, middle, and bottom, where the top and the bottom ROIs contained the respective cranial and caudal growth plates while the middle ROI was the region with/without the lesion. Each ROI was divided in 9 subregions of interest (subROIs). The lesion mainly involved one single subROI, but adjacent subROIs might be affected, as well. Histograms were reported for each ROI of the intact vertebrae and of those with lesions to evaluate how the lesion affected the strain distribution in the different regions of the bone.

Thematic maps [42] were used to show regional differences in ε_p3_ absolute values in the same subROIs, before and after the introduction of the lesion: positive differences were reported in red, negative differences were reported in blue, differences below the sum of the systematic and random errors for the ε_p3_ (within measurement uncertainties, see S1 Appendix for the detailed values of the uncertainties for each specimen) were reported in grey.

## Results

All vertebrae in the intact condition were loaded up to 6500N without visible permanent damage in the microCT images. After the lesion, specimens #1, #4 and #5 did not fracture at 6500N load, whereas specimens #2 and #3 failed at 3000N and 4500N, respectively.

**Fig 3:**
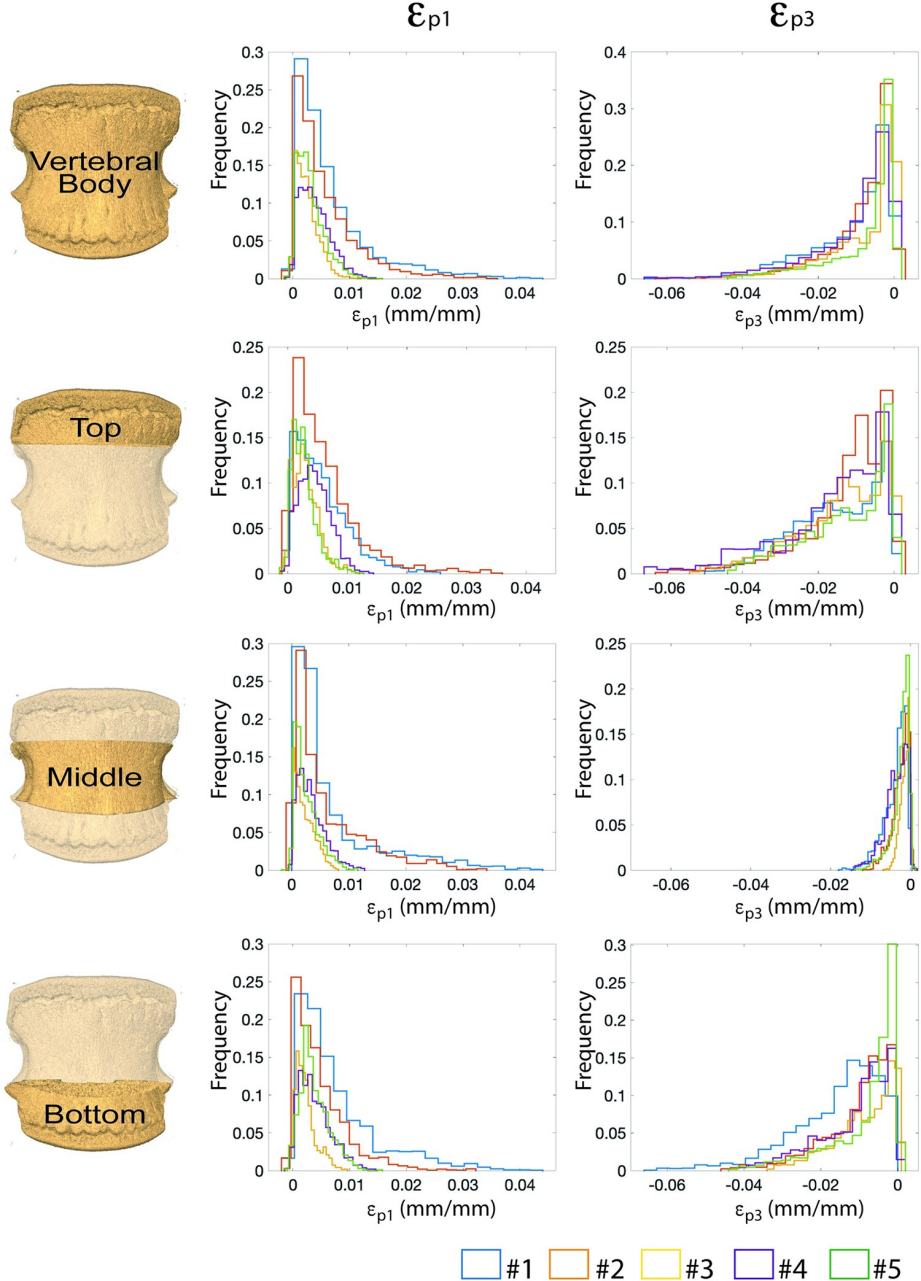
Frequency plot for the ε_p1_ and ε_p3_ evaluated for each intact specimen for the entire vertebra, for the top, middle and bottom ROIs.

The values of principal strains within the intact vertebral bodies showed consistent distributions for all specimens, with similar median values of ε_p1_ (0.22% to 0.46%) and ε_p3_ (-0.72% to -0.33%). Similar median strain values among the specimens were found also in the different ROIs for ε_p1_ (0.28% to 0.48% in the top ROI; 0.19% to 0.39% in the middle ROI; and 0.19% to 0.53% in the bottom ROI) and ε_p3_ (-1.44% to -0.96% in the top ROI; -0.32% to -0.12% in the middle ROI; and -1.28% to -0.47% in the bottom ROI) (Fig 3). Nevertheless, higher peaks of ε_p1_ in every region were found for two specimens (#1 and #2) compared to the others.

**Fig 4:**
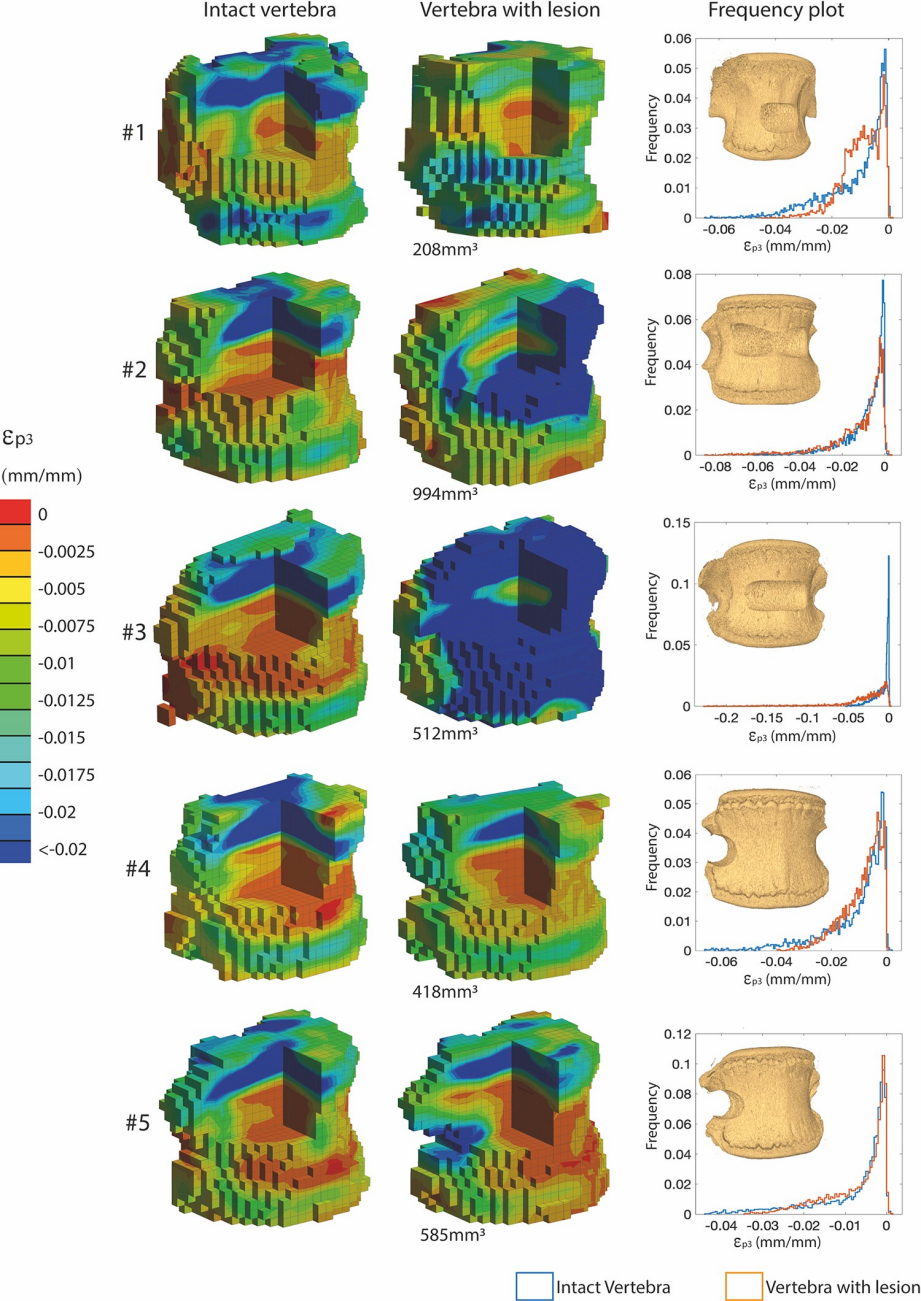
ε_**p3**_ **distributions in the intact vertebrae (left) and in the vertebrae with artificial lesions (middle).** The volume of the lesion is reported close to the strain distributions for vertebrae with defects. On the right, rendering of the vertebra with the created artificial lesion and frequency plots for ε_p3_ in the whole vertebral body with (orange) and without (blue) the lesions are reported. For the intact vertebrae, the larger strains localized in correspondence of the growth plates, both on the top and the bottom. For the vertebrae with the artificial lesions, the strain distribution was different and strain concentrations were visible around the lesion.

**Fig 5:**
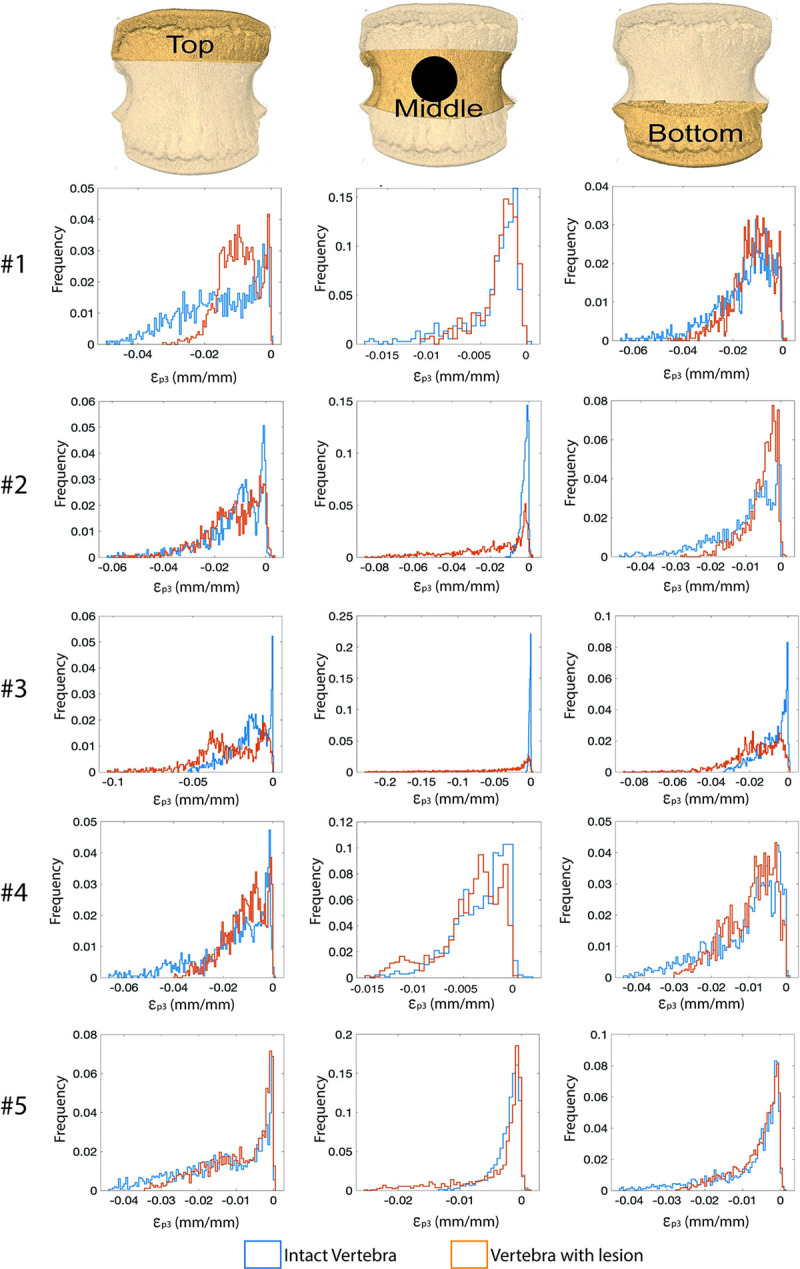
ε_**p3**_ **distributions were evaluated in the top (left column), middle (central column), and bottom (right column) longitudinal ROIs.** The blue histograms refer to intact specimens, the orange ones to those with lesions.

The distributions of ε_p3_ (Fig 4) within the intact vertebral bodies showed similar patterns with larger deformations (ε_p3_ <-2%) localized in correspondence of the top and bottom ROIs, that include the growth plates, and quite uniform strains (ε_p3_ of approximately -0.25%) in the middle of the vertebral body. While the ε_p3_ over the vertebral body reached peaks of approximately -6%, localised in the top or bottom ROIs, the peak deformation in the middle ROI was lower (approximately -1.8%) (Fig 5).

The tests on the vertebrae with lesions (volume between 208mm^3^ and 994mm^3^, corresponding to the 0.8% and 4.6% of the volume of the vertebral body, Fig 4) showed an increase of the strain in proximity of the lesion and a decrease of the strain in the growth plate regions. For the specimens with the artificial lesion that did not fail under 6500N the peak of ε_p3_ within the whole vertebral body reached approximately -4%. Conversely, the specimens that failed showed larger ε_p3_, approximately -8% and -20%, for the specimen #2 and #3, respectively. In both cases the strain concentration, and the consequent failure, localised close to the lesion, in the midplane of the vertebral body (Fig 4).

The strain distributions changed consistently (except for specimen #3 that failed before 6500N load) due to the lesion, with decreased deformations in the top and bottom ROIs and increased deformations in the middle ROI with respect to the intact condition (Fig 5). Different variations in strain values before and after the creation of the defect were found for the different lesions. Lower increases in deformation were induced by lateral lesions compared to those observed for anterior lesions which largely compromised the vertebra.

**Fig 6:**
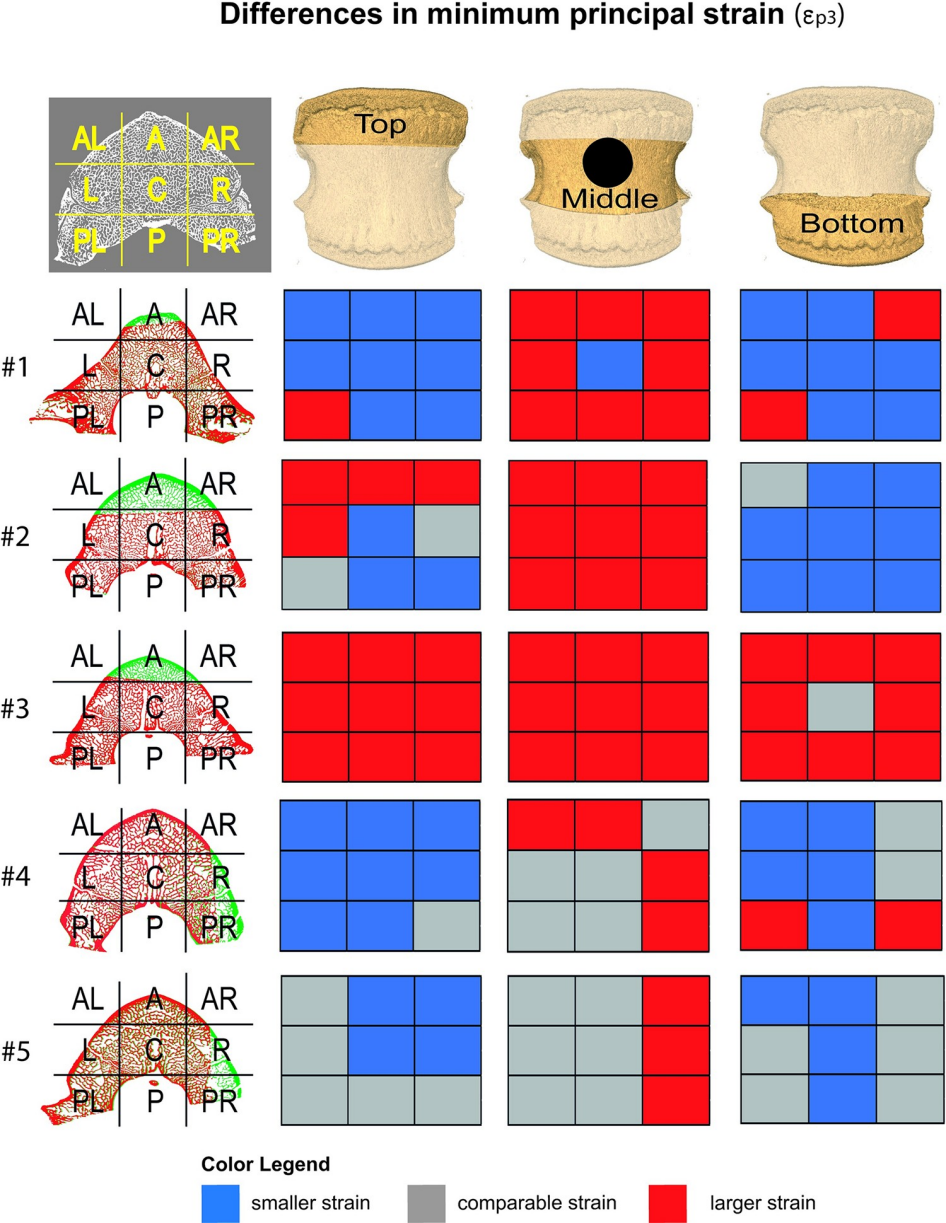
Changes in the strain values at 6500N (exception for #2 and #3) for each subROI with and without the lesion. The first column represents the scheme of the different subROIs above the central cross-sections before (green) and after (red) the lesion. The lesion mainly affected one single subROI, but may also involve adjacent subROI. Increase (red boxes) or decrease (blue boxes) of ε_p3_ in the top (second column), middle (third column) and bottom (forth column) ROIs are reported on the right. Differences below the DVC measurement uncertainties are reported in grey.

The number of nodes in each subROI varied from 45 (“AL” subROI in the middle ROI of the specimen #3) to 378 (“C” subROI, in the middle ROI of the specimen #1). The differences in subROIs strain, before and after the introduction of the lesions, confirmed the observations on the ROIs: increased strains were observed in most compartments of the middle ROIs, with higher values in the regions adjacent to the created lesions (Fig 6). Conversely, a homogeneous decrease of the strain in most regions of the top and bottom ROIs were observed for specimens #1, #4 and 5#. Exceptions were observed for the failed specimens (#2 and #3) for which an increase of the strain was observed in all regions (#3) or in both middle ROI and most of the top ROI (#2).

## Discussion

The aim of this study was to develop a novel methodological approach to experimentally evaluate the effect of artificially induced lesions on the strain field inside the vertebral bodies tested in compression. An analysis to evaluate the differences in the strain pattern before and after the lesion was proposed for the whole vertebral body and in specific compartments.

Most studies in the literature that have analysed the effects of lytic metastasis on the spine stability were performed with artificial lesions and simplified loading conditions (i.e.: compression or a combination of compression and flexion). While reduction in apparent strength and stiffness [3, 6, 8] and redistribution in the strain over the anterior surface [4, 5, 9] were observed, no detailed analysis in the internal regions of the vertebral bodies was performed, limiting our understanding of the effects of the lesion on the vertebral mechanical competence. Just one study used the DVC approach to study the biomechanics of the vertebrae with actual metastasis [10]. They showed the potentiality of the DVC in this field, the work was performed on rat vertebrae, with sensible different dimensions and cortical/trabecular bone ratio with respect to human vertebrae. To overcome this limitation, in this study a method based on *in situ* mechanical testing, microCT imaging and DVC analysis was developed to test porcine vertebrae, and a protocol was developed to identify changes in local strains over the entire vertebral body or in different compartments, due to a focal lesion.

The robustness of the method was confirmed by the low measurement uncertainties, in line with previously reported results for different bone types and image resolutions [31], and the similar strain conditions obtained in five intact vertebrae (small differences could be due to the different vertebral levels), with strain concentrations in the caudal and cranial regions that include growth plates (top and bottom ROIs), and lower strain in the middle portion of the vertebral body. The method was able to quantify the localization of the strain in the different regions (i.e. growth plates, lesions, intact bone), highlighting its potential for analysing the biomechanical properties of vertebrae with heterogeneous material properties (e.g. vertebrae with real lesions or with large osteophytes, vertebrae from subjects not yet skeletally mature). The observed higher peaks of maximum principal strains for two specimens could be due to differences in the vertebral properties (size, shape and microstructure), and in the alignment with respect to the applied load. A number of other DVC approaches have been recently developed [43–46] and the developers are welcome to use the provided images to highlight the potential of their method.

The creation of artificial focal lesions dramatically redistributed the strain pattern for all specimens, reducing the localised strain concentrations in the growth plates. In some cases, the lesion reduced dramatically the strength of the vertebra and led to failure. This could be explained considering that the lesions were in the region (mid height of the transverse plane) with the higher shell load fraction, and that the ratio of the removed cortical/trabecular bone can affect the failure [47]. In the two vertebrae with lesions that failed before the target load (6500N), the algorithm allowed to identify the fracture location that corresponded to regions with higher strain. Moreover, reduced strains were observed in the regions of the growth plates, that may be due to a redistribution of the loads transferred to the endplates through the intervertebral discs on the vertebra, as a consequence of a reduced stiffness of the middle portion of the vertebra. Additional studies should be performed to better understand and interpretate the behaviour of the growth plates [48].

This study has some limitations. First, the method was proposed on porcine vertebrae, of different spinal levels for their low inter-variability compared to human specimens. In fact, the porcine vertebrae have a denser tissue compared to human vertebrae (bone volume fraction: range for porcine 33–48%, range for human 3–23%; trabecular separation: range for porcine 396–503 micrometers, range for human 600–2000 micrometers) [17, 49]. While this choice is acceptable for setting the procedure and showing the potential of the approach, further studies on a large number of human vertebrae must be performed for evaluating the effect of lesion position and size on the vertebral mechanical properties, thereby providing useful experimental evidence for future clinical applications. Due to the differences between the porcine and human vertebral microstructure, DVC parameters specific for the studied microstructure should be identified [31], as described in the S1 Appendix. Moreover, the growth plates in the porcine vertebrae are still open. The DVC approach highlighted that this anatomical feature affects the strain patterns within the porcine vertebral body and, as the growth plates are closed in adult human vertebrae, this difference should be taken into account when using a porcine vertebra for spine research [20, 50–53]. The limited space in the microCT scanner required the removal of the posterior arches. While the removal of this anatomical feature influenced the physiological load sharing along the spine segment, the DVC approach aimed at evaluating different strain distributions before and after the induction of the lesion in the vertebral body under compression. Nevertheless, in future studies where more complex loading scenarios are used, the posterior arches should not be removed when possible. In addition, the loading conditions and loading rate were far from the expected physiological ones. Furthermore, a small number of specimens was tested in order to perform detailed DVC assessments. This, in addition to the former limitations, prevents from generalizing the effect of the metastatic features, which was not the aim of the study. Nevertheless, the obtained findings were sufficient to demonstrate the robustness of the entire procedure. The induced lesions, used in this study and in similar studies for vertebrae [5] and femurs [25], were far from real metastases, which may affect the tissue within and around the border of the lesion. In this study only one lesion shape was induced to define and test the proposed methodology. Finally, this approach was performed using high resolution microCT scans, not applicable in clinics. A similar approach could be applied in the future to clinical CT images [54], but higher measurement uncertainty should be expected [31].

In conclusion, the proposed experimental methods based on DVC measurements allowed to identify the changes in the 3D strain distribution inside the vertebral body associated to the introduction of artificial lytic-like lesions. This procedure, once applied to a large number of human vertebrae with a factorial design of the metastatic features, can improve our understanding of the effect of lesion properties on the vertebral failure behaviour and validate FE models of vertebrae with artificial lesions or real metastases.

## Supporting information

S1 Appendix Measurement uncertainty and loading magnitude. The procedure to identify the best compromise between the measurement uncertainty and the measurement spatial resolution is reported. In addition, preliminary study to select the most relevant loading magnitude.
(PDF)

S2 Appendix Maximum principal strain. The maximum principal strain maps and the histograms for each specimen, both intact and with lesions, are reported.
(PDF)
