# Supplementary material for: A novel approach to evaluate the effects of artificial bone focal lesion on the three-dimensional strain distributions within the vertebral body
Source: PLoS One. 2021 Jun 1;16(6):e0251873. doi: 10.1371/journal.pone.0251873 (PMC8168867; doi:10.1371/journal.pone.0251873)
Supplement: S1 Appendix — The procedure to identify the best compromise between the measurement uncertainty and the measurement spatial resolution is reported. In addition, preliminary study to select the most relevant loading magnitude. (PDF) [file pone.0251873.s001.pdf]

Supporting Information 1 (S1 appendix)

Preliminary tests were performed on the specimen #1 to optimise the DVC parameters with uncertainties analyses in zero-strain condition for different values of the nodal spacing [1]. Once the optimal DVC nodal spacing was chosen, the uncertainties were also evaluated for each specimen acquiring repeated scans, in zero-strain conditions. Finally, the specimen was tested under different loading magnitudes in order to choose the optimal load to use for the other specimens.

Identification of the optimal nodal spacing

A preliminary test was performed on the specimen #1 to find the optimal nodal spacing (NS) to be used in the DVC approach, by finding the best compromise between uncertainties (an order of magnitude below the strain associated to physiological load, i.e. 1500-3000 microstrain) and spatial resolution of the method [2]. Two repeated scans (ScanA vs ScanB) of the whole specimen under 50N preload (zero-strain condition) were acquired without any repositioning. The average (Mean Absolute Error, MAER) and the standard deviation (Standard Deviation of the Error, SDER) of the average of the absolute values of the six component of strain in each node, were evaluated for NS values equal to 10, 30, 50, 70 and 90 voxel [3].

A nodal spacing of 50 voxels was associated to MAER of 227 $\mu\varepsilon$ and SDER of 148 $\mu\varepsilon$ (Fig. S1), and was chosen for further DVC analyses.

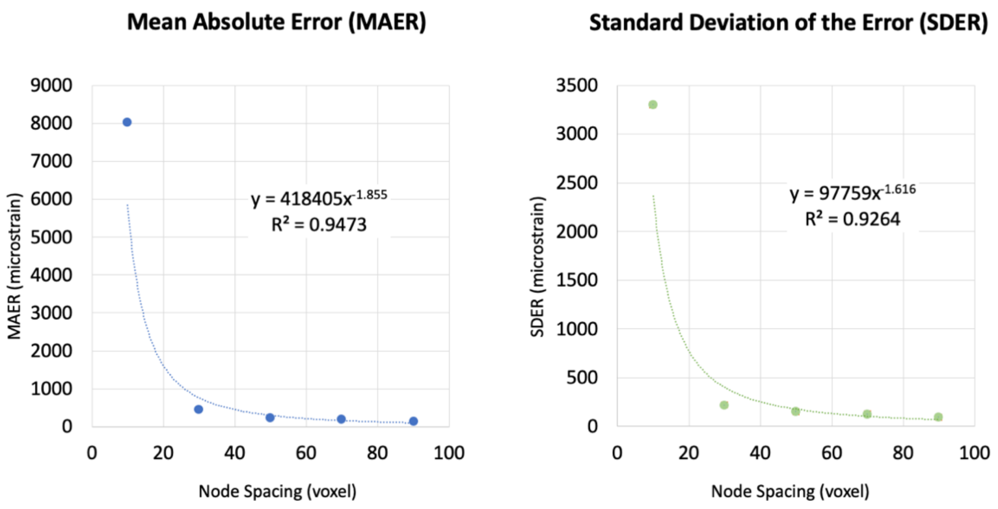

Fig. S1 Mean Absolute Error (MAER) and Standard Deviation of the Error (SDER) evaluated on the whole specimen #1 through the repeated scans in zero-strain (ScanA vs ScanB). The dimension of the isotropic voxel was 39 micrometres.

Measurement uncertainties related to each specimen

The measurement uncertainties were evaluated for each specimen acquiring two repeated scans, in zero-strain conditions, of a reduced portion of the vertebral body (Scan1-pre and Scan1-crop acquired in the middle portion of the vertebral body, both under 50N preload as described in the manuscript and in the Fig. 2). A NS equal to 50 voxels was used for the DVC uncertainties.

The systematic (average) and random (standard deviation) errors of the minimum principal strains (ε_{p3}), as well as the MAER and SDER were computed for each specimen.

The average of the systematic error and the random error (Table S1) of the ε_{p3} in the middle portion of the vertebral body were -623 $\mu\varepsilon$ and 435 $\mu\varepsilon$, respectively.

Table S1: For each specimen the systematic error and the random error for ε_{p3} were computed in the middle portion of the vertebral body under 50N preload. The mean average error (MAER) and the standard deviation of the error (SDER) are also reported.

**Specimen**	**Systematic error** [$\mu\varepsilon$]	**Random error** [$\mu\varepsilon$]	**MAER** [$\mu\varepsilon$]	**SDER** [$\mu\varepsilon$]
#1	-168	85	161	125
#2	-660	475	462	331
#3	-582	363	418	303
#4	-566	402	419	247
#5	-1139	852	932	681
**Mean**	**-623**	**435**	**478**	**337**
**St. Dev.**	**346**	**276**	**280**	**208**

The measurement uncertainties under full load (6500N) were not evaluated in this study. A similar study was conducted on mouse tibia using the same DVC approach [4]. Strain uncertainties similar to those in zero-strain were evaluated.

Moreover, for other bone structures scanned with similar image quality (femoral heads scanned with the same scanning protocol [5]), similar uncertainties were found if tested on

repeated virtually deformed images: the bone is scanned twice in its preloaded condition and a virtual known affine deformation is applied to the second scan. Considering that similar uncertainties can be expected between these two applications we have not performed a similar test here.

Definition of the load magnitude

In addition, the specimen #1 was stepwise loaded and microCT scanned under an axial compressive load equal to 1.5kN, 3kN, 4kN and 6.5kN, in order to identify the optimal load to generate relevant strains within the porcine vertebrae without damaging the tissue. The DVC approach was applied with NS equal to 50 voxels, and the principal strains (ε_{p1}, ε_{p2} and ε_{p3}) were calculated across the whole specimen.

The best load compromise was found for an axial compressive load of 6.5kN (Fig. S2). In fact, the strain values were large enough to reduce the effect of the DVC measurement uncertainties and it did not generate any visible damage in the microCT images of the vertebral body.

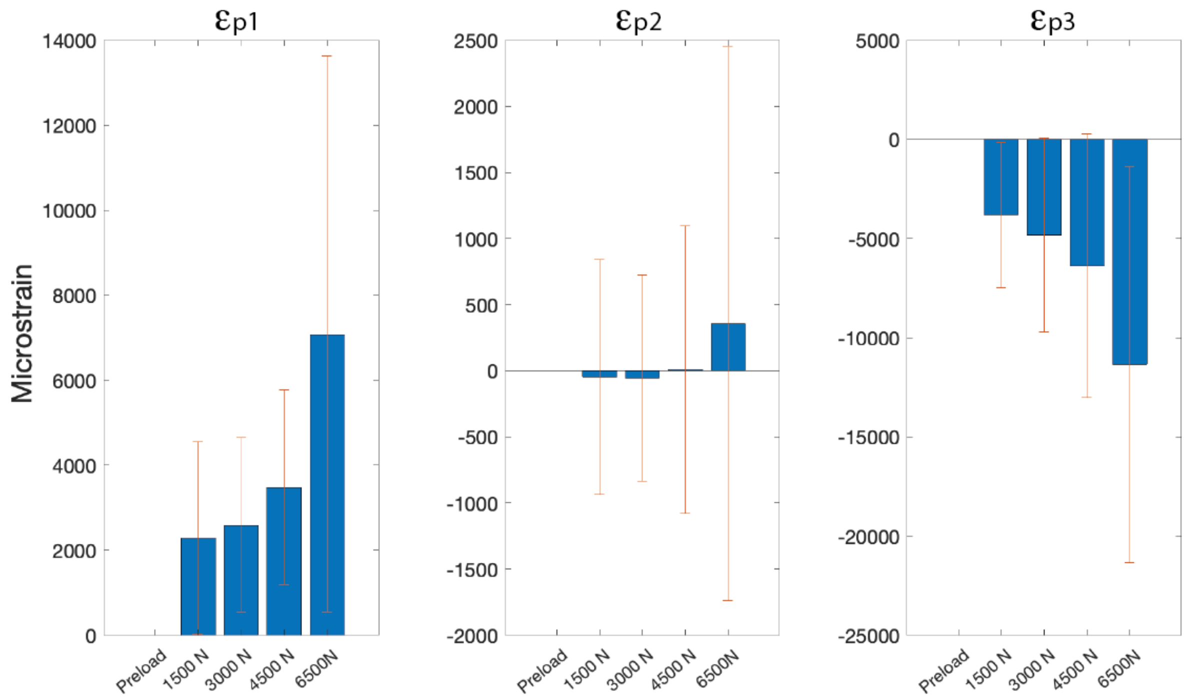

Fig. S2: The mean (blue bars) and the standard deviation (red error bars) of the ε_{p1}, ε_{p2} and ε_{p3} evaluated within the vertebral body of the intact vertebra #1 evaluated for each load.

Bibliography

1. Liu L, Morgan EF. Accuracy and precision of digital volume correlation in quantifying displacements and strains in trabecular bone. Journal of Biomechanics. 2007;40: 3516–3520. doi:10.1016/j.jbiomech.2007.04.019

2. Dall'Ara E, Barber D, Viceconti M. About the inevitable compromise between spatial resolution and accuracy of strain measurement for bone tissue: A 3D zero-strain study. Journal of Biomechanics. 2014;47: 2956–2963. doi:10.1016/j.jbiomech.2014.07.019

3. Palanca M, Cristofolini L, Dall'Ara E, Curto M, Innocente F, Danesi V, et al. Digital volume correlation can be used to estimate local strains in natural and augmented vertebrae: An organ-level study. Journal of Biomechanics. 2016;49: 3882–3890. doi:10.1016/j.jbiomech.2016.10.018

4. Giorgi M, Dall'Ara E. Variability in strain distribution in the mice tibia loading model: A preliminary study using digital volume correlation. Medical Engineering & Physics. 2018;62: 7–16. doi:10.1016/j.medengphy.2018.09.001

5. Ryan MK, Oliviero S, Costa MC, Wilkinson JM, Dall'Ara E. Heterogeneous Strain Distribution in the Subchondral Bone of Human Osteoarthritic Femoral Heads, Measured with Digital Volume Correlation. Materials. 2020;13: 4619. doi:10.3390/ma13204619
