# Supplementary material for: A novel approach to evaluate the effects of artificial bone focal lesion on the three-dimensional strain distributions within the vertebral body
Source: PLoS One. 2021 Jun 1;16(6):e0251873. doi: 10.1371/journal.pone.0251873 (PMC8168867; doi:10.1371/journal.pone.0251873)
Supplement: S2 Appendix — The maximum principal strain maps and the histograms for each specimen, both intact and with lesions, are reported. (PDF) [file pone.0251873.s002.pdf]

Supporting Information 2 (S2 appendix)

The ε_{p1} maps showed, as for the ε_{p3}, a consistent strain distribution for the specimens tested before the artificial lesion, with the strain in a range approximately 0 to 4% (Fig. S3). The lesions changed the strain distribution with a trend of increased ε_{p1} for most specimens except specimen #1 which showed a reduction of strain. Nevertheless, similar median strain values were observed.

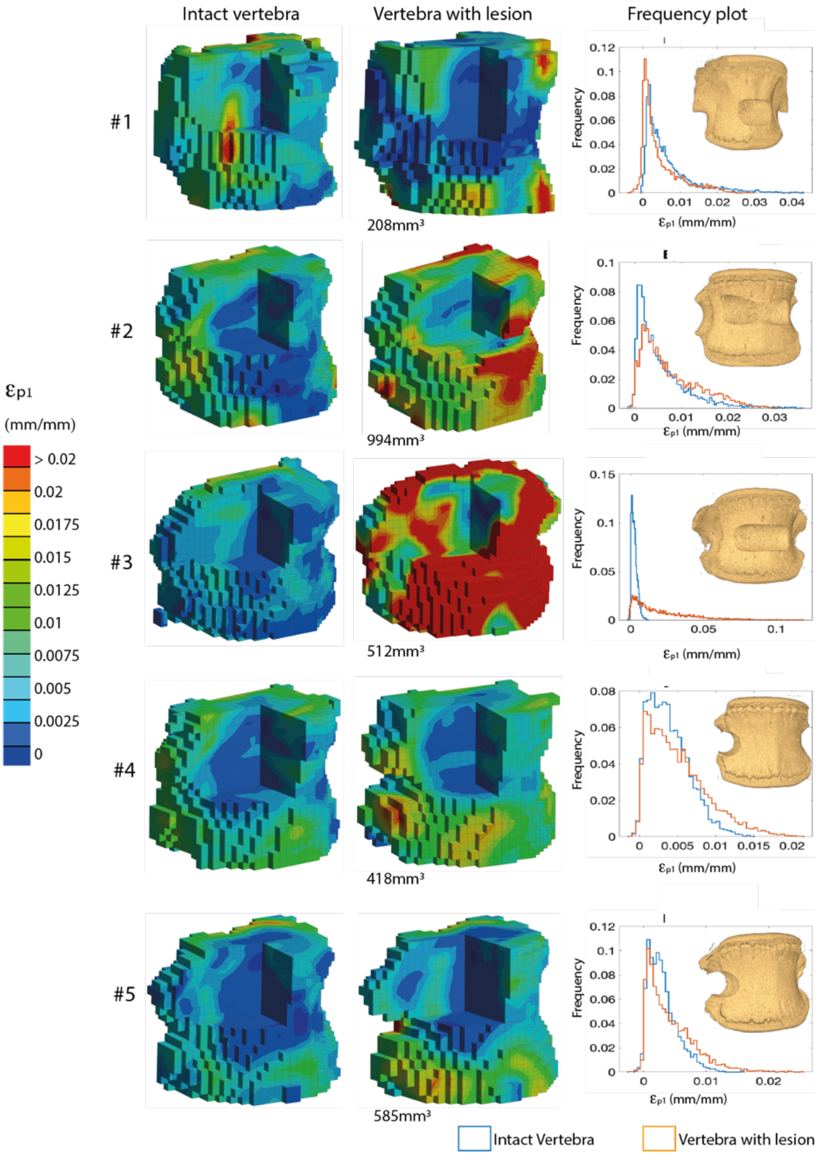

Fig. S3: ε_{p1} distributions in the intact vertebrae (left) and in the vertebrae with artificial lesions (middle). Size of the induced lesion is reported close to the strain distributions for vertebrae with defects. On the right, rendering of the vertebra with the created artificial lesion and frequency plots for ε_{p3} in the whole vertebral body with and without the lesions are reported.
